# Short-term facilitation of breathing upon cessation of hypoxic challenge is impaired in male but not female endothelial NOS knock-out mice

**DOI:** 10.1038/s41598-021-97322-3

**Published:** 2021-09-15

**Authors:** Paulina M. Getsy, Sripriya Sundararajan, Walter J. May, Graham C. von Schill, Dylan K. McLaughlin, Lisa A. Palmer, Stephen J. Lewis

**Affiliations:** 1 Department of Pediatrics, Biomedical Research Building BRB 319, Case Western Reserve University, 10900 Euclid Avenue Mail Stop 1714, Cleveland, OH 44106-1714 USA; 2 Department of Physiology and Biophysics, Case Western Reserve University, Cleveland, OH USA; 3 Pediatric Respiratory Medicine, University of Virginia School of Medicine, Charlottesville, VA USA; 4 Department of Pharmacology, Case Western Reserve University, Cleveland, OH USA; 5 Functional Electrical Stimulation Center, Case Western Reserve University, Cleveland, OH USA; 6 Present Address: Division of Neonatology, Department of Pediatrics, University of Maryland School of Medicine, Baltimore, MD 21201 USA

**Keywords:** Neuroscience, Physiology, Systems biology

## Abstract

Decreases in arterial blood oxygen stimulate increases in minute ventilation via activation of peripheral and central respiratory structures. This study evaluates the role of endothelial nitric oxide synthase (eNOS) in the expression of the ventilatory responses during and following a hypoxic gas challenge (HXC, 10% O_2_, 90% N_2_) in freely moving male and female wild-type (WT) C57BL6 and eNOS knock-out (eNOS–/–) mice. Exposure to HXC caused an array of responses (of similar magnitude and duration) in both male and female WT mice such as, rapid increases in frequency of breathing, tidal volume, minute ventilation and peak inspiratory and expiratory flows, that were subject to pronounced roll-off. The responses to HXC in male eNOS–/– mice were similar to male WT mice. In contrast, several of the ventilatory responses in female eNOS–/– mice (e.g., frequency of breathing, and expiratory drive) were greater compared to female WT mice. Upon return to room-air, male and female WT mice showed similar excitatory ventilatory responses (i.e., short-term potentiation phase). These responses were markedly reduced in male eNOS–/– mice, whereas female eNOS–/– mice displayed robust post-HXC responses that were similar to those in female WT mice. Our data demonstrates that eNOS plays important roles in (1) ventilatory responses to HXC in female compared to male C57BL6 mice; and (2) expression of post-HXC responses in male, but not female C57BL6 mice. These data support existing evidence that sex, and the functional roles of specific proteins (e.g., eNOS) have profound influences on ventilatory processes, including the responses to HXC.

## Introduction

Signaling from peripheral and central neural structures combine to monitor ventilation in response to changes in arterial blood-gas chemistry^1–5^. Glomus cells in the carotid body respond to decreases in blood pO_2_, increases in blood pCO_2_ and decreases in blood pH, which results from carbonic anhydrase-catalyzed production of protons from enhanced bioavailability of circulating CO_2_^6–13^. Upon activation, the glomus cells release neurotransmitters^14–24^ that excite closely apposed chemoafferent nerve terminals of the carotid sinus nerve (CSN) which synapse in the commissural nucleus tractus solitarius (cNTS)^6–13^. The input from the CSN chemoafferents triggers a series of cardiorespiratory reflexes (e.g., increases in minute ventilation) in order to restore arterial blood gas homeostasis^10,17,25^. The roles of carotid body chemoafferents in eliciting the ventilatory responses to hypoxic (HXC) and hypercapnic (HCC) gas challenges have been explored in humans^26–34^, and a variety of experimental animals including, dogs^35–37^, rats^38–53^ and mice^54–56^. The expression of hypoxic ventilatory responses (e.g., increases in minute ventilation) are vitally dependent upon the carotid body-CSN complex, whereas the expression of hypercapnic ventilatory responses are dependent on both the carotid body-CSN complex and CO_2_/H^+^–sensitive neurons within the retrotrapezoid nucleus and parafacial complex within the brainstem medulla obongata^57–66^.

Wild-type and genetically-engineered mice are widely used to investigate mechanisms by which HXC elicits carotid body *dependent* and *independent* ventilatory responses^23,24,56,67–81^. To date only one study has addressed the role of the carotid body-CSN complex in the ventilatory responses to hypercapnia^50^. In that study, ventilatory responses of urethane-anesthetized adult male C57BL/6CrSlc mice to hypoxia (e.g., 10% O_2_, 90% N_2_) and hyperoxic (100% O_2_)-hypercapnia (5% CO_2_) gas challenges were recorded before and after bilateral CSN transection (CSNX). The authors found that the ventilatory responses elicited by hypoxia and by hyperoxic-hypercapnia were diminished after bilateral CSNX. Moreover, we have published similar results suggesting that the ventilatory responses elicited by HXC are markedly reduced in freely moving adult male C57BL6 mice with bilateral CSNX^56^. In addition, it is well documented that the increases in breathing seen in response to HXC and HCC often remain present even after exposure to the challenges has ended (i.e., upon return to room-air). This pattern of responses is collectively referred to as short-term potentiation (STP) of ventilation^56,82–85^. In C57B6 mice, in particular, it is even more remarkable that the ventilatory responses during HXC show dramatic roll-off (i.e., ventilatory parameters return toward pre-HXC values) and the return to room-air results in an immediate and relatively sustained (for over 10 min) elevation in the frequency of breathing, for example, among many other changes to ventilatory parameters^56,73–76^. Importantly, these post-HXC responses are not associated with abnormal behaviors, but are absent in mice with bilateral CSN transection, and thereby most likely to be due to an increase in CSN chemoafferent nerve activity^56^.

In this study, we used the C57BL6 mouse strain because it is widely used to study the physiology of cardiorespiratory systems, and it has been used previously to generate genetically-engineered mice to study mechanisms involved in cardiorespiratory control processes^56,74,75,86–89^. The C57BL6 strain is also used by sleep-apnea researchers because it displays irregular breathing patterns, including apneas and sighs, during sleep and wakefulness, and markedly disordered breathing upon return to room-air after hypoxic exposures^76,90–97^. Therefore, many of the neurochemical^87,94,98–103^ and genetic^86,91–93,95,104–107^ factors underlying the breathing patterns of C57BL6 mice, and their responses to hypoxic gas exposures, are known. In addition, the potential role of structural differences in the carotid bodies of C57BL6 mice has also been studied^108–110^.

Over the past couple decades scientists have investigated the roles of neuronal NOS (nNOS) and eNOS in the expression of the ventilatory responses to hypoxic (HXC) and hypercapnic (HCC) gas challenges, and the responses that occur upon return to room-air, in anesthetized and freely moving mice^70,84,85,111,112^. In these studies, they used adult male and female nNOS–/– and eNOS–/– mice that were derived from hybrids of 129/SV and C57BL6 strains, and used the eNOS and nNOS hybrids as wild-type (WT) controls. With respect to the freely moving mice, the studies found that (1) the ventilatory responses to HXC were enhanced in nNOS–/– mice compared to WT mice^70,111^, (2) the ventilatory responses to HXC were reduced in eNOS–/– mice^70^, (3) the ventilatory responses during HCC in nNOS–/– and eNOS–/– mice were similar to those in WT mice^70,111,112^, and (4) short-term potentiation of ventilatory parameters after HXC was virtually absent in nNOS–/– mice^84,85^ with no report if eNOS–/– mice were studied. Of importance was that Kline and colleagues made no mention as to whether female WT, nNOS–/– and eNOS/- mice had different responses to those reported for males^70,84,85,111,112^. Moreover, it should be noted that in addition to the studies mentioned above, eNOS–/– mice of hybrid 129/SV and C57BL6 background^113–117^ have been used extensively to study the physiological roles of this NOS isoform.

The objective of the present study was to compare the ventilatory and thermoregulatory responses that occur during and following a 15 min HXC (10% O_2_, 90% N_2_) in adult male and female C57BL6 mice and in eNOS–/– mice of C57BL6 background. It should be noted that these male and female mice have also been used extensively to study the physiological roles of this NOS isoform^78–118,126^, in addition to eNOS–/– mice of hybrid 129/SV and C57BL6 background^113–117^. Our data show that the ventilatory responses in male eNOS–/– mice during a HXC were similar to those of their WT male counterparts, whereas several of the ventilatory responses in female eNOS–/– mice (e.g., frequency of breathing) were greater than in their female WT counterparts. In addition, the ventilatory responses recorded upon return to room-air (i.e., STP phase) in the male eNOS–/– mice were markedly reduced compared to male WT mice, whereas the STP responses in female eNOS–/– mice were very similar to those of the female WT mice. Our studies complement those of Kline and colleagues^70,84,85,111,112^ and suggest that sex and genetic factors may play a role in determining the importance of eNOS in the ventilatory responses that occur during and following hypoxic exposures.

## Methods

### Permissions

All procedures were performed in accordance with the National Institute of Health (NIH) “Guide for the Care and Use of Laboratory Animals” (NIH Publication No. 80–23) revised in 1996 (https://www.nap.edu/catalog/5140/guide-for-the-care-and-use-of-laboratory-animals). In addition, all studies were carried out in compliance with the ARRIVE (Animal Research: Reporting of In Vivo Experiments) guidelines (http://www.nc3rs.org.uk/page.asp?id=1357). The protocols were approved by the Animal Care and Use Committees of the University of Virginia and Case Western Reserve University.

### Mice

This study used adult male and female homozygous eNOS–/– mice (C57BL/6 J-NOS3tm1Unc) with C57BL/6 J (C57BL6) genetic background and age-matched male and female C57BL6 mice as controls. All mice were purchased from Jackson Laboratory (Bar Harbor, ME, USA). Mice were delivered pathogen free and housed under pathogen free conditions with a 12 h light–dark cycle. The status of the menstrual cycle in the female mice was not taken into account because its effect on respiration and the ventilatory responses to HXC is minor, and female mice responsiveness in the luteal phase is enhanced by 5–10% compared to the follicular phase^127,128^, with no correlation apparent between the ratio of progesterone and estradiol and the HXC response^129^.

### Whole body plethysmography

On the day of the experimental study, each freely moving mouse was placed in an individual whole body plethysmographs (Buxco® Small Animal Whole Body Plethysmography, DSI a division of Harvard Biosciences, Inc., St. Paul, MN, USA) to record ventilatory parameters continuously as detailed previously^56,73–76^. The ventilatory parameters were chosen to give in-depth analyses of the differences in resting breathing patterns and responses to HXC in the mice^56,73–76,102,130,131^. Directly recorded parameters were (a) frequency of breathing (Freq), (b) tidal volume, (TV) (c) inspiratory time (Ti, duration of inspiration) and expiratory time (Te, duration of expiration), and (d) peak inspiratory flow (PIF) and peak expiratory flow (PEF). The calculated parameters were (a) minute ventilation (MV = Freq x TV), and (b) inspiratory drive (TV/Ti) and expiratory drive (TV/Te). Provided software (Fine Pointe, BUXCO) constantly corrected digitized values for changes in chamber temperature and humidity. A rejection algorithm was included in the breath-by-breath analysis to exclude episodes of nasal breathing. Pressure changes associated with the respiratory waveforms were converted to volumes (TV, PIF, PEF) using algorithms of Epstein and colleagues^76^. Factoring in chamber temperature and humidity, the cycle analyzers filtered the acquired signals, and proprietary algorithms (Fine Pointe, BUXCO) generated box flow data that identified a breath. From that data vector, minimum and maximum values were determined. The flows at this point were "box flow" signals. From this array, the minimum and maximum box flow values were then determined. The minimum and maximum box flows were multiplied by the compensation factor provided by the selected algorithm, producing the TV, PIF and PEF parameters. All of the plethysmography and body temperature studies were performed in a quiet laboratory with relative humidity of 49 ± 2% and room temperature of 21.3 ± 0.2 °C.

### Hypoxic gas challenge in plethysmography chambers

The male and female WT (C57BL6) and eNOS–/– mice were placed in individual plethysmography chambers and allowed 45–60 min to acclimatize to the chambers. The mice were then exposed to a HXC (10% O_2_, 90% N_2_) for 15 min and then they were re-exposed to room-air for another 15 min.

### Body temperature recordings during and after HXC

Substantial changes in body temperature can influence whole body plethysmography analyses of the respiratory waveforms although these have to be dramatic (over 6 °C) for the data produced by the in-built software algorithms not to be accurate (https://www.datasci.com/products/buxco-respiratory-products/finepointe-whole-body-plethysmography). As such, because we were interested in how the absence of eNOS affects body temperature responses during and following HXC, and because of the technical need to establish the status of body temperature, we determined body temperature as described below. Note that the temperature of the hypoxic chamber was maintained at 21.1 ± 0.3 °C before and during establishing the hypoxic environment, a temperature similar to that of the room-air in the laboratory (21.3 ± 0.2 °C, P > 0.05, chamber temperature *versus* room temperature). This study used (1) male C57BL6 mice (n = 6, 84.7 ± 0.6 days of age, 25.2 ± 0.2 g body weight); (2) male eNOS–/– (n = 6, 84.2 ± 0.6 days of age, 24.8 ± 0.3 g body weight); (3) female C57BL6 mice (n = 6, 85.7 ± 0.9 days of age, 19.6 ± 0.2 g body weight); and (4) female eNOS–/– (n = 6, 86.0 ± 0.4 days of age, 19.2 ± 0.3 g body weight). There were no differences in body weights between the C57BL6 and eNOS–/– mice for males or females (P > 0.05, for all comparisons). The mice and thermistor probe (YS 451, Yellow Springs Instruments, Yellow Springs, OH), which was connected to a battery-operated thermometer unit (YSI 400) and used to measure and record colonic body temperature of the mice, were placed in a Hypoxic Work Station (Coy, Grass Lake, MI), which had room-air flowing through the housing chamber. This chamber allowed access to the mice and recording equipment via air-tight arm holes. After 5 min, the thermistor probe was inserted 1.5–2.0 cm into the rectum of each mouse to record core body temperature. The mice were removed and a hypoxic environment (10% O_2_, 90% N_2_) was established in the chamber using a Pro:Ox : Model 350 unit (Biospherix, Lacona, NY).

The mice were returned to the chamber once the hypoxic environment was established and body temperature was recorded 5 and 15 min during the HXC and 5 and 15 min after return to room-air (induced rapidly by fully opening the chamber). The mice were returned to the hypoxic chamber through a small door in the side of the chamber. This process was very quick (approximately 2 s) and elicited negligible changes in O_2_ concentration in the chamber as directly monitored by the inbuilt O_2_ sensor. It is important to note, that this procedure did not involve undue restraint of the mice, or any procedure deemed overtly stressful. However, it is certainly possible that the return to the chamber disturbed the mice, although by the time of the first recording (i.e., at 5 min) it seemed likely that the temperature recordings were accurate in that they were not affected by returning the mice to the chambers.

### Statistics

Several steps were taken to determine the total responses during HXC and upon return to room-air. Specifically, for each mouse, we determined the mean of the 20 values recorded during the 5 min period (4 values per min) immediately prior to exposing the mice to the HXC. These mean values were used to determine the arithmetic changes (e.g., actual value at the first time-point minus the mean of the pre-value) during the 5 min of HXC (20 arithmetic change values) and upon return to room-air including the first 5 min (20 arithmetic change values) and the entire 15 min (60 arithmetic change values) of this phase. The individual arithmetic responses were then simply added together to provide the sum of the responses. The mean and SEM of the individual data were then determined. All summary data included those in the Tables are presented as mean ± SEM.

The data were analyzed by one-way or two-way analysis of variance^132^ followed by Student’s modified t-test with Bonferroni corrections for multiple comparisons between means using the error mean square terms from each ANOVA^133–135^. A value of P < 0.05 denoted the initial level of statistical significance that was modified according to the number of comparisons between means as detailed by Wallenstein et al.^133^ The modified t-statistic is t = (mean of group 1—mean of group 2)/[s x (1/n_1_ + 1/n_2_)^1/2^] where s^2^ = the mean square within groups term from the ANOVA (the square root of this value is taken for the modified t-statistic formula) and n_1_ and n_2_ are the numbers of mice in each group that are being compared. Based on an elementary inequality called Bonferroni’s inequality, a conservative critical value for the modified t-statistic taken from tables of t-distribution using a significance level of P/m, where m is the number of comparisons between groups to be performed. The degrees of freedom are those for the mean square for within-group variation from ANOVA tables^132^. In most cases, the critical Bonferroni value cannot be obtained from conventional tables of the t-distribution but may be approximated from widely available tables of the normal curve by t* = z + (z + z3)/4n, with n being the degrees of freedom and z being the critical normal curve value for P/m^132,133^. Wallenstein et al^133^ first reported that the Bonferroni procedure is preferable for general use since it is easiest to apply, has the widest range of applications, and gives critical values that are lower than those of other procedures if the investigator can limit the number of comparisons, and that will be only slightly larger than those of other procedures if many comparisons are made. The practical application of the Bonferroni procedure demonstrated by Wallenstein et al^133^ was confirmed and expanded upon in separate studies by Ludbrook^134^ and by McHugh^135^. A value of P < 0.05 was taken as the initial level of statistical significance^133–135^.

**Figure 1:**
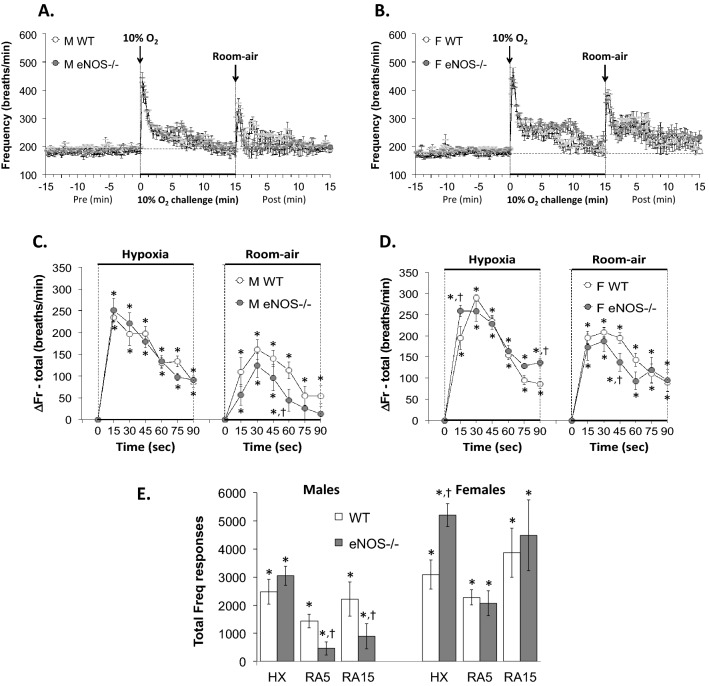
Panels (**A**) and (**B**): Frequency of breathing (Fr, Freq) values before, during a hypoxic gas challenge (HXC, 10% O_2_, 90% N_2_), and upon return to room-air in male (M) and female (F) wild-type (WT) and eNOS knock-out (eNOS–/–) mice. Panels (**C**) and (**D**): Arithmetic changes in Freq in WT and eNOS–/– male and female mice during the first 90 s of exposure to the HXC and the first 90 s upon return to room-air. Panel (**E**): Total changes in Freq in WT and eNOS–/– male and female mice during the HXC and during the first 5 min (RA5) and entire 15 min (RA15) return to room-air. The data are shown as mean ± SEM. The data were analyzed by one-way or two-way ANOVA followed by Student’s modified t-test with Bonferroni corrections for multiple comparisons between means using the error mean square terms from each ANOVA. *P < 0.05, significant response. ^†^*P* < 0.05, eNOS–/– *versus* WT within each sex.

**Figure 2:**
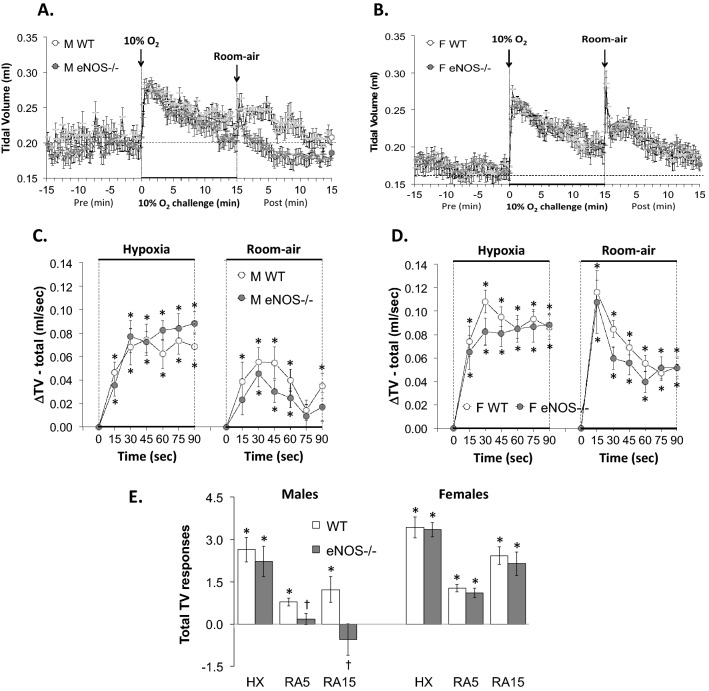
Panels (**A**) and (**B**): Tidal volume (TV) values before, during a hypoxic gas challenge (HXC, 10% O_2_, 90% N_2_), and upon return to room-air in male (M) and female (F) wild-type (WT) and eNOS knock-out (eNOS–/–) mice. Panels (**C**) and (**D**): Arithmetic changes in TV in WT and eNOS–/– male and female mice during the first 90 s of exposure to the HXC and the first 90 s upon return to room-air. Panel (**E**): Total changes in TV in WT and eNOS–/– male and female mice during HXC and during the first 5 min (RA5) and entire 15 min (RA15) return to room-air. The data are shown as mean ± SEM. The data were analyzed by one-way or two-way ANOVA followed by Student’s modified t-test with Bonferroni corrections for multiple comparisons between means using the error mean square terms from each ANOVA. *P < 0.05, significant response. ^†^*P* < 0.05, eNOS–/– *versus* WT within each sex.

**Figure 3:**
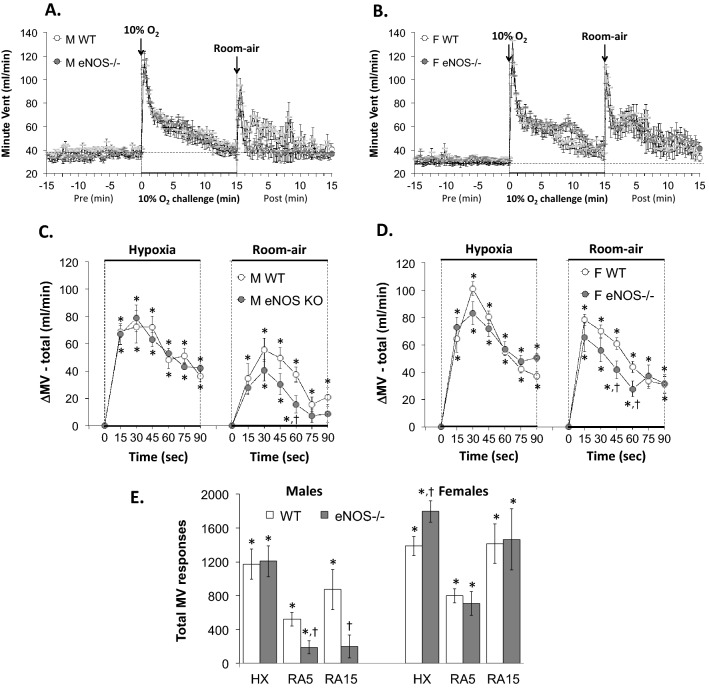
Panels (**A**) and (**B**): Minute Ventilation (MV) values before, during a hypoxic gas challenge (HXC, 10% O_2_, 90% N_2_), and upon return to room-air in male (M) and female (F) wild-type (WT) and eNOS knock-out (eNOS–/–) mice. Panels (**C**) and (**D**): Arithmetic changes in MV in WT and eNOS–/– male and female mice during the first 90 s of exposure to the HXC and the first 90 s upon return to room-air. Panel (**E**): Total changes in MV in WT and eNOS–/– male and female mice during HXC and during the first 5 min (RA5) and entire 15 min (RA15) return to room-air. The data are shown as mean ± SEM. The data were analyzed by one-way or two-way ANOVA followed by Student’s modified t-test with Bonferroni corrections for multiple comparisons between means using the error mean square terms from each ANOVA. *P < 0.05, significant response. ^†^P < 0.05, eNOS–/– *versus* WT within each sex.

**Figure 4:**
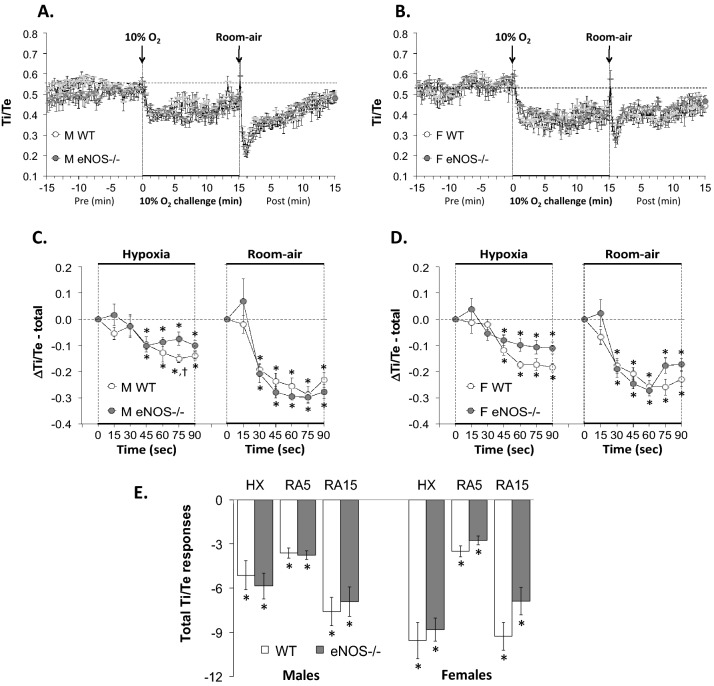
Panels (**A**) and (**B**): Inspiratory time/Expiratory time (Ti/Te) ratios before, during a hypoxic gas challenge (HXC, 10% O_2_, 90% N_2_), and upon return to room-air in male (M) and female (F) wild-type (WT) and eNOS knock-out (eNOS–/–) mice. Panels (**C**) and (**D**): Arithmetic changes in Ti/Te ratios in WT and eNOS–/– male and female mice during the first 90 s of exposure to the HXC and the first 90 s upon return to room-air. Panel (**E**): Total changes in Ti/Te in WT and eNOS–/– male and female mice during HXC and during the first 5 min (RA5) and entire 15 min (RA15) return to room-air. The data are shown as mean ± SEM. The data were analyzed by one-way or two-way ANOVA followed by Student’s modified t-test with Bonferroni corrections for multiple comparisons between means using the error mean square terms from each ANOVA. *P < 0.05, significant response. ^†^P < 0.05, eNOS–/– *versus* WT within each sex.

**Figure 5:**
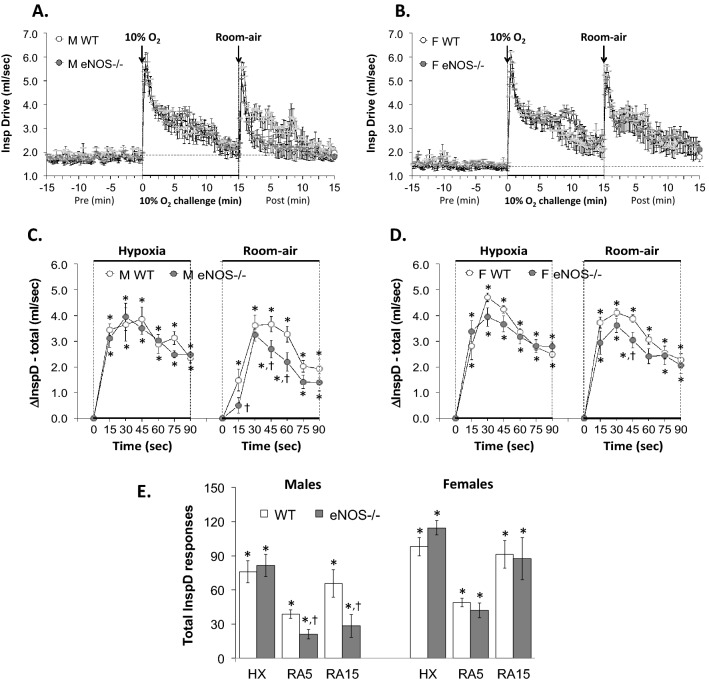
Panels (**A**) and (**B**): Inspiratory drive (InspD, TV/Ti) values before, during a hypoxic gas challenge (HXC, 10% O_2_, 90% N_2_), and upon return to room-air in male (M) and female (F) wild-type (WT) and eNOS knock-out (eNOS–/–) mice. Panels (**C**) and (**D**): Arithmetic changes in TV/Ti in WT and eNOS–/– male and female mice during the first 90 s of exposure to the HXC and the first 90 s upon return to room-air. Panel (**E**): Total changes in TV/Ti in WT and eNOS–/– male and female mice during HXC and during the first 5 min (RA5) and entire 15 min (RA15) return to room-air. Data are shown as mean ± SEM. Data were analyzed by one-way or two-way ANOVA followed by Student’s modified t-test with Bonferroni corrections for multiple comparisons between means using the error mean square terms from each ANOVA. *P < 0.05, significant response. ^†^P < 0.05, eNOS–/– *versus* WT within each sex.

**Figure 6:**
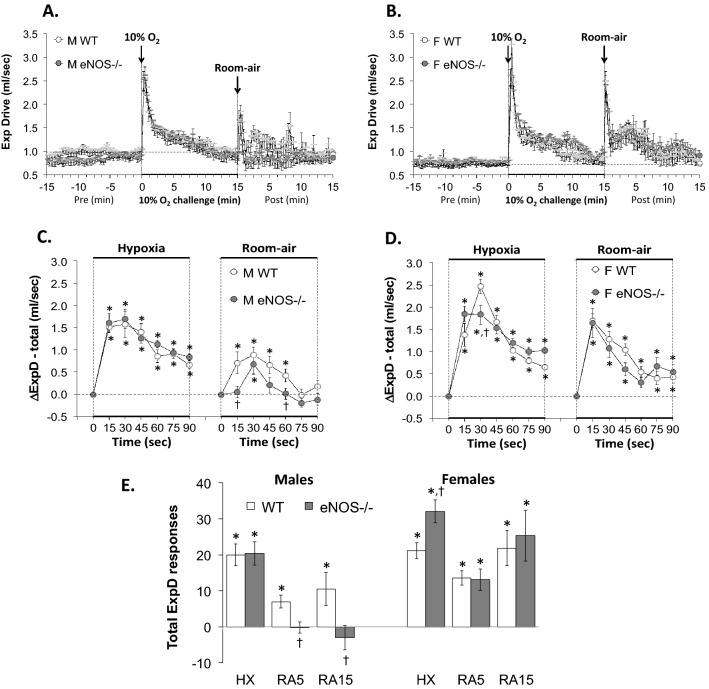
Panels (**A**) and (**B**): Expiratory drive (ExpD, TV/Te) values before, during a hypoxic gas challenge (HXC, 10% O_2_, 90% N_2_), and upon return to room-air in male (M) and female (F) wild-type (WT) and eNOS knock-out (eNOS–/–) mice. Panels (**C**) and (**D**): Arithmetic changes in TV/Te in WT and eNOS–/– male and female mice during the first 90 s of exposure to the HXC and the first 90 s upon return to room-air. Panel (**E**): Total changes in TV/Te in WT and eNOS–/– male and female mice during the HXC and during the first 5 min (RA5) and entire 15 min (RA15) return to room-air. The data are shown as mean ± SEM. The data were analyzed by one-way or two-way ANOVA followed by Student’s modified t-test with Bonferroni corrections for multiple comparisons between means using the error mean square terms from each ANOVA. *P < 0.05, significant response. ^†^P < 0.05, eNOS–/– *versus* WT within each sex.

**Figure 7:**
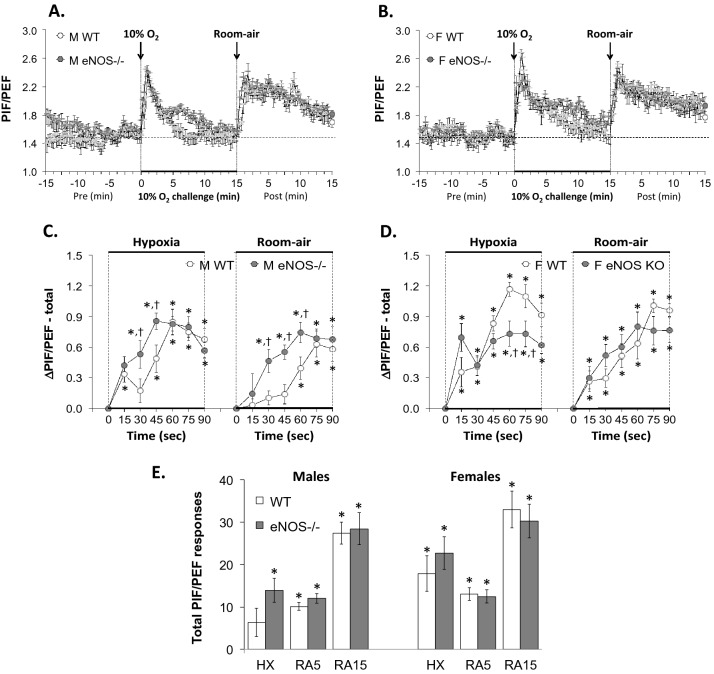
Panels (**A**) and (**B**): Peak Inspiratory Flow/Peak Expiratory Flow (PIF/PEF) ratios before, during a hypoxic gas challenge (HXC, 10% O_2_, 90% N_2_), and upon return to room-air in male (M) and female (F) wild-type (WT) and eNOS knock-out (eNOS–/–) mice. Panels (**C**) and (**D**): Arithmetic changes in PIF/PEF ratios in WT and eNOS–/– male and female mice during the first 90 s of exposure to the HXC and the first 90 s upon return to room-air. Panel (**E**): Total changes in PIF/PEF ratios in WT and eNOS–/– male and female mice during HXC and during the first 5 min (RA5) and entire 15 min (RA15) return to room-air. Data are shown as mean ± SEM. The data were analyzed by one-way or two-way ANOVA followed by Student’s modified t-test with Bonferroni corrections for multiple comparisons between means using the error mean square terms from each ANOVA. *P < 0.05, significant response. ^†^P < 0.05, eNOS–/– *versus* WT within each sex.

With respect to Panel C in Figs. 1, 2, 3, 4, 5, 6 and 7 and Supplemental Figs. 3–6,, the data from the male mice were subjected to a nested repeated measures (two-way) ANOVA design to allow for six between-group comparisons between the WT and eNOS–/– mice during the HXC (i.e., 15, 30, 45, 60, 75 and 90 s) and six between group comparisons for the subsequent room-air phase (i.e., 15, 30, 45, 60, 75 and 90 s) via Student's modified t-test with Bonferroni corrections for multiple comparisons between means using the error mean square term from the ANOVAs, as defined by Winer^132^ and Wallenstein et al.^133^ The data from the female mice in Panel D of Figs. 1–7 and Supplemental Figs. 3–6 were analyzed separately in a similar fashion. With respect to Panel E of Figs. 1–7 and Supplemental Figs. 3–6, the total responses of the male and female WT and eNOS–/– mice recorded during the HXC and subsequent return to room-air were subjected to a repeated measures ANOVA with Bonferroni correction for multiple comparisons (i.e., 6 in total) between the WT and eNOS–/– groups of mice^133–135^.

## Results

### Ages, body weights and baseline values in WT and eNOS–/– mice

The general information about the mice used in the plethysmography study and their resting ventilatory parameters recorded over the 15 min period immediately before exposure to the HXC are presented in Table 1. With respect to the male mice, the ages and body weights of the WT and eNOS–/– mice were similar to one another, as were all of the recorded and calculated ventilatory parameters. With respect to the female mice, there were no between-group differences in the ages, body weights or resting ventilatory parameters between the WT and eNOS–/– mice. The only statistically significant difference was that the weights of the female mice were smaller than those of the respective male mice.

**Table 1:** Resting parameters in wild-type and endothelial nitric oxide synthase knock-out mice.

Parameter	Male		Female	
	WT	eNOS–/–	WT	eNOS–/–
Number of mice	12	13	12	12
Age, days	78.0 ± 0.2	80.8 ± 2.2	79.0 ± 0.2	76.0 ± 2.2
Body weight, g	24.9 ± 0.5	23.7 ± 0.6	19.1 ± 0.3*	18.2 ± 0.4‡
Frequency, breaths/min	186 ± 8	185 ± 6	182 ± 4	172 ± 3
Tidal volume, ml	0.205 ± 0.005	0.196 ± 0.009	0.167 ± 0.006	0.169 ± 0.004
Minute ventilation, ml/min	37.4 ± 1.3	35.6 ± 1.3	29.9 ± 0.8	29.0 ± 1.1
Inspiratory time, sec	0.115 ± 0.002	0.113 ± 0.003	0.120 ± 0.002	0.126 ± 0.002
Expiratory time, sec	0.227 ± 0.007	0.225 ± 0.008	0.226 ± 0.007	0.240 ± 0.005
Inspiratory time/expiratory time	0.520 ± 0.016	0.509 ± 0.011	0.543 ± 0.014	0.538 ± 0.012
Inspiratory drive, ml/sec	1.82 ± 0.08	1.75 ± 0.07	1.41 ± 0.04	1.36 ± 0.05
Expiratory drive, ml/sec	0.92 ± 0.03	0.88 ± 0.03	0.75 ± 0.02	0.72 ± 0.03
Peak inspiratory flow (PIF), ml/sec	2.97 ± 0.14	2.89 ± 0.12	2.29 ± 0.06	2.28 ± 0.08
Peak expiratory flow (PEF), ml/sec	1.96 ± 0.09	1.90 ± 0.0.10	1.57 ± 0.07	1.50 ± 0.05
PIF/PEF	1.53 ± 0.05	1.56 ± 0.06	1.49 ± 0.05	1.53 ± 0.03

The data are presented as mean ± SEM. WT, wild-type C57BL6 mice. eNOS–/–, endothelial nitric oxide synthase knock-out mice. There were no differences in ages, body weights and resting ventilatory parameters between male WT and eNOS–/– mice and between female WT and eNOS–/– mice (*P < 0.05, body weights, male WT *versus* female WT mice; and ‡*P* < 0.05, body weights, male eNOS–/– *versus* female eNOS–/– mice).

### Changes in body temperature

The changes in body temperature recorded before, during, and after HXC in the male and female WT and eNOS–/– mice are summarized in Table 2. All of the male and female mice had equivalent body temperatures to one another. The body temperatures of all mice decreased approximately 0.4 °C after 15 min exposure to HXC, but rapidly recovered to baseline upon return to room-air. There were no between group differences at any of the time points.

**Table 2:** Changes in body temperature during a hypoxic gas challenge and upon return to room-air.

Sex	Group	Pre	Arithmetic changes in body temperature (^o^C)			
			Hypoxia		Room-air	
			5 min	15 min	5 min	15 min
Male	WT	36.6 ± 0.1	−0.05 ± 0.07	−0.35 ± 0.11*	−0.10 ± 0.13	+ 0.02 ± 0.06
	eNOS–/–	36.8 ± 0.1	−0.08 ± 0.08	−0.45 ± 0.10*	−0.03 ± 0.08	+ 0.05 ± 0.04
Female	WT	36.8 ± 0.1	+ 0.02 ± 0.07	−0.38 ± 0.06*	−0.07 ± 0.08	+ 0.03 ± 0.09
	eNOS–/–	37.1 ± 0.1	0.00 ± 0.06	−0.42 ± 0.07*	−0.02 ± 0.07	+ 0.13 ± 0.07

The data are presented as mean ± SEM. There were 6 mice in each group. (*P < 0.05, significant change from Pre). Note, there were no between group differences at any time point (*P* > 0.05, for all comparisons).

### Comparisons of ventilatory responses to HXC in male and female WT mice

The arrays of ventilatory responses that occurred during HXC and upon return to room-air in male and female WT mice are described below. It is important to note that all of the ventilatory responses elicited by the HXC in male and female WT mice were qualitatively and quantitatively similar to one another. In addition, the total increases in Freq, TV and MV that occurred upon return to room-air were greater in the female WT mice compared to the male WT mice at both the 5 and 15 min time points (Figs. 1, 2 and 3), whereas the total increases in all other parameters that occurred upon return to room-air were similar in male and female WT mice at both the 5 and 15 min time points (Figs. 4–7 and Supplemental Figs. 3–6).

### Ventilatory responses during and following HXC in WT and eNOS–/–mice

#### General comments

Panels A and B of Figs. 1–7 and Supplemental Figs. 3–6 display the raw values recorded before, during HXC and upon return to room-air in the male WT and eNOS–/– and in the female WT and eNOS–/– mice, respectively. With respect to Panels C-E of Figs. 1–7 and Supplemental Figs. 3–6, the data from male WT and eNOS–/– mice were analyzed by two-way ANOVA and Student's modified t-test with Bonferroni corrections for multiple comparisons between means using the error mean square term from the ANOVA^133–135^. The female WT and eNOS–/– mice were analyzed by the same procedures. As mentioned above, the data are presented as mean ± SEM. However, for comparison, the data for frequency of breathing, tidal volume and minute ventilation in male mice is expressed as mean ± standard deviation (SD) in Supplemental Figs. 1 and 2.

#### Frequency of breathing (Fr, Freq)

As seen in Fig. 1, HXC elicited robust initial increases in Freq in male and female WT mice that were, as expected, subject to pronounced roll-off (panels A and B, respectively). The rate of reaching the peak increases in Freq (panel C) during HXC and the total (arithmetic) responses (panel E) were similar in male WT and eNOS–/– mice. The rate of reaching the peak increases in Freq during HXC was similar in female WT and eNOS–/– mice (panel D), however, the total responses were significantly greater in the female eNOS–/– mice than in female WT mice (panel E). Upon return to room-air, Freq values returned to baseline somewhat more rapidly in the male eNOS–/– mice than male WT mice (panels A and C), and the total responses over the 5 and 15 min periods were significantly smaller in the male eNOS–/– mice than in male WT mice (panel E, columns RA5 and RA15). The Freq responses recorded upon return to room-air at the 5 and 15 min periods were similar in female eNOS–/– mice and female WT mice (panels B, D and E).

#### Tidal Volume (TV)

As seen in Fig. 2, HXC elicited robust initial increases in TV in male and female WT mice that were, as expected, subject to roll-off (panels A and B, respectively). The rate of reaching the peak increases in TV (panels C and D) during HXC and the total (arithmetic) responses (panel E) were similar between male WT and eNOS–/– mice and female WT and eNOS–/– mice. Upon return to room-air, TV values returned toward baseline at a similar initial rate (over the course of 90 s) in the male WT and eNOS–/– mice (panels A and C). However, the total responses upon return to room-air at the 5 and 15 min periods were significantly lower in male eNOS–/– mice than in male WT mice (panel E, columns RA5 and RA15). The TV responses recorded upon return to room-air in female eNOS–/– mice were similar to those in female WT mice (panels B, D and E).

#### Minute ventilation (MV)

As seen in Fig. 3, HXC elicited robust initial increases in MV in male and female WT mice that displayed pronounced roll-off (panels A and B, respectively). The rate of reaching the peak increases in MV (panels C and D) during the HXC and the total (arithmetic) responses (panel E) were similar in male WT and eNOS–/– mice. The rate of reaching the peak increases in MV during HXC was similar in female WT and eNOS–/– mice (panel D), except for the 15 s time-point in female eNOS–/– mice (i.e., a greater decrease in Ti than that observed in female WT mice) (panel D). Additionally, the total responses were significantly greater in female eNOS–/– mice compared to female WT (panel E). Upon return to room-air, MV returned to baseline somewhat more rapidly in the male eNOS–/– mice than male WT mice (panels A and C), and the total responses upon return to room-air at the 5 and 15 min periods were significantly lower in the male eNOS–/– mice (panel E, columns RA5 and RA15). The MV total responses recorded upon return to room-air in the female eNOS–/– mice were similar to those in the WT females (panels B, D and E).

#### Inspiratory time (Ti)

As seen in Supplemental Fig. 3, HXC elicited robust initial decreases in Ti in male and female WT mice that were, as expected, subject to pronounced roll-off (panels A and B, respectively). The rate of reaching the peak decreases in Ti (panels C and D) during the HXC and the total (arithmetic) responses (panel E) were similar between male WT and male eNOS–/– mice. The rate of reaching peak decreases in Ti during HXC was similar in female WT and eNOS–/– mice (panel D), however the total responses were significantly greater in female eNOS–/– mice compared to female WT (panel E). Upon return to room-air, the changes in Ti (an initial decrease followed by gradual recovery toward baseline) were essentially similar in all mice (panels A-E) at the 5 and 15 min periods, except for the 15 s time-point in male eNOS–/– mice (i.e., no change from baseline in contrast to the decrease in Ti seen in male WT mice) (panel C).

#### Expiratory time (Te)

As seen in Supplemental Fig. 4, HXC elicited robust initial decreases in Te in male and female WT mice that were, as expected, subject to pronounced roll-off (panels A and B, respectively). The rate of reaching the peak decreases in Te (panels C and D) during the HXC and the total (arithmetic) responses (panel E) were similar between male WT and male eNOS–/– mice. The rate of reaching the peak decreases in Te during HXC was faster in female eNOS–/– mice compared to female WT mice (Panel D). The decrease in Te seen in the total responses (panel E) during HXC was greater in female eNOS–/– mice than WT mice. Upon return to room-air, Te more or less returned to baseline levels in the male WT mice, but displayed robust increases in the male eNOS–/– mice (panels A, C and E). Upon return to room-air, in the female mice, the WT and eNOS–/– showed similar initial falls in Te and then recovery to baseline (panels B, D and E).

#### Inspiratory time/expiratory time (Ti/Te)

As seen in Fig. 4, HXC elicited similar decreases in Ti/Te ratios in male and female WT mice and male and female eNOS–/– mice (panels A-D, respectively). The decrease in Ti/Te ratio was due to the relatively greater decrease in Te than Ti (Supplemental Figs. 3 and 4). Upon return to room-air, Ti/Te ratios fell initially (again because of relatively greater reductions in Te than Ti), and then gradually recovered toward baseline levels (panels A-D, respectively). There were no significant changes in Ti/Te ratios in WT male mice compared to eNOS–/– male mice and WT female mice compared to eNOS–/– female mice during HXC or return to room-air (panel E).

#### Inspiratory drive (InspD, TV/Ti)

As seen in Fig. 5, HXC elicited robust initial increases in TV/Ti in male and female WT and eNOS–/– mice that displayed pronounced roll-off (panels A and B, respectively). The rate of reaching the peak increases in inspiratory drive during the HXC and the total (arithmetic) responses (panel E) were similar between male WT and eNOS–/– mice and female WT and eNOS–/– mice (panels C and D, respectively). Upon return to room-air, inspiratory drive returned to baseline more rapidly in the male eNOS–/– mice compared to the male WT mice (panels A and C) and the total responses at the 5 and 15 min periods were significantly smaller in the male eNOS–/– mice compared to the WT male mice (panel E, columns RA5 and RA15). The inspiratory drive total responses recorded upon return to room-air in female eNOS–/– mice were essentially similar to those in WT female mice, except for the 45 s time-point in which the female eNOS–/– mice displayed a significantly smaller total inspiratory drive response compared to female WT mice (panels B, D and E).

#### Expiratory drive (ExpD, TV/Te)

As seen in Fig. 6, HXC elicited robust initial increases in TV/Te in male and female WT and eNOS–/– mice that displayed pronounced roll-off (panels A and B, respectively). The rate of reaching peak increases in expiratory drive (panels C and D) during the HXC and the total (arithmetic) responses (panel E) were similar between male WT and eNOS–/– mice. The rate of reaching peak increases in expiratory drive (panel D) during the HXC in female eNOS–/– mice was essentially similar to that in WT females, except for the 30 s time-point in which the female eNOS–/– mice displayed a significantly smaller total expiratory drive response compared to female WT mice. Additionally, the total expiratory drive responses were greater in the female eNOS–/– mice compared to WT female mice (panel E). Upon return to room-air, expiratory drive returned to baseline faster in the male eNOS–/– mice compared to the male WT mice (panels A and C) and thus the total responses at the 5 and 15 min periods were smaller in male eNOS–/– mice (panel E, columns RA5 and RA15). The expiratory drive total responses recorded upon return to room-air in female eNOS–/– mice were similar to those in WT female mice (panels B, D and E).

#### Peak inspiratory flow (PIF)

As seen in Supplemental Fig. 5, HXC elicited robust initial increases in PIF in male and female WT and eNOS–/– mice that displayed pronounced roll-off (panels A and B, respectively). The rate of reaching peak increases in PIF (panels C and D) during HXC and the total (arithmetic) responses (panel E) were similar between male WT and eNOS–/– mice and female WT and eNOS–/– mice. Upon return to room-air, the initial increases in PIF in male eNOS–/– mice were essentially similar to those in the male WT mice (panels A and C), but the total responses at the 15 min time-point were smaller in male eNOS–/– mice compared to WT male mice (panel E, column RA15). The PIF total responses recorded upon return to room-air in female eNOS–/– mice were essentially similar to those in WT female mice, except for the 45 s time-point in which the female eNOS–/– mice displayed a significantly smaller total PIF response compared to female WT mice. (panels B, D and E).

#### Peak expiratory flow (PEF)

As seen in Supplemental Fig. 6, HXC elicited robust initial increases in PEF in male and female WT and eNOS–/– mice that displayed pronounced roll-off (panels A and B, respectively). The rate of reaching peak increases in PEF (panels C and D) during the HXC and the total (arithmetic) responses (panel E) were similar between male WT and eNOS–/– mice and female WT and eNOS–/– mice. Upon return to room-air, the initial increases in PEF in the male eNOS–/– mice occurred faster than in the male WT mice (panels A and C), and the total responses at the 5 and 15 min periods were significantly smaller in male eNOS–/– mice than in male WT mice (panel E, columns RA5 and RA15). Upon return to room-air, the initial increases in PEF in the female eNOS–/– mice occurred slower than in the female WT mice (panels B and D), although the total PEF responses were similar between female eNOS–/– mice and female WT mice (panel E, columns RA5 and RA15).

#### Peak inspiratory flow/ peak expiratory flow (PIF/PEF)

As seen in Fig. 7, HXC elicited robust initial increases in PIF/PEF ratios in male and female WT and eNOS–/– mice that displayed pronounced roll-off (panels A and B, respectively). The increase in PIF/PEF ratio was due to a relatively greater increase in PIF than PEF during HXC (Supplemental Figs. 5 and 6). The initial increases in PIF/PEF ratios occurred at slightly different rates in the four groups of mice (panels C and D) during the HXC, but the total responses were similar between the male WT and male eNOS–/– mice and female WT and female eNOS–/– mice (panel E). Upon return to room-air, the initial increases in PIF/PEF occurred faster in male eNOS–/– mice than the male WT mice (panels A and C), but the total responses at the 5 and 15 min periods were similar in male eNOS–/– and male WT mice (panel E, columns RA5 and RA15). The initial increases in PIF/PEF ratios and the total responses recorded upon return to room-air in female eNOS–/– mice were similar to those in female WT mice (panels B, D and E).

## Discussion

The present study demonstrates that resting ventilatory parameters in male and female eNOS–/– mice are similar to their respective male and female WT control mice of C57BL6 background. As such, it appears that eNOS is not essential for the maintenance of respiratory timing (e.g., Freq, Ti and Te), ventilatory mechanics (e.g., TV, PIF and PEF), and the products of timing and mechanics, including MV and inspiratory and expiratory drives. These findings on eNOS–/– mice of C57BL6 background support data reported by Kline et al^112^ who reported that (1) resting respiratory rate, phrenic nerve activity and minute neural respiration of urethane-anesthetized eNOS–/– mice were similar to their WT controls, which consisted of hybrids of 129/SV and C57BL6 mice; and (2) resting Freq, TV and MV of awake eNOS–/– mice were similar to WT mice. In agreement with the findings of other groups^74,76,90–97^, our male and female C57BL6 mice displayed pronounced roll-off during the HXC. The mechanisms by which roll-off occurs during hypoxic challenge in C57BL6 mice have received considerable attention^74,76,90–97^. These mechanisms involve alterations in mitochondrial activity and the activities of oxygen/ATP-dependent enzymes due to the hypoxic exposure, and changes in the central (e.g., retrotrapezoid nucleus) chemoreceptor CO_2_/H^+^-sensing pathways as a result of the progressive hypocapnia that develops during the HXC. The changes in roll-off in the male and female eNOS–/– mice will be discussed below.

Additional key findings of our study include that (1) the ventilatory responses during HXC in male eNOS–/– mice (including rate of rise and total response) were similar to those in the WT male mice, and (2) some of the ventilatory responses of female eNOS–/– were exaggerated compared to female WT mice. More specifically, the total increases in Freq (and total decreases in Ti and Te), MV and expiratory drive were greater in female eNOS–/– mice than in the female WT mice during the HXC. These data are in contrast to those of Kline et al^112^ who reported that male and female eNOS–/– mice of 129/SV and C57BL6 background displayed diminished ventilatory responses to hypoxic challenge (12% O_2_, 88%N_2_) compared to the WT hybrids. Kline et al^112^ also reported that cGMP levels (an index of nitric oxide bioactivity) in the brainstem of eNOS–/– mice rose more than in the brainstem of WT mice. As such, the diminished hypoxic ventilatory responses of eNOS–/– with hybrid 129/SV and C57BL6 background may involve the loss of responsiveness of carotid body chemoafferent input to the brainstem and overproduction of nitric oxide in the brainstem (presumably from neuronal NOS), which has a negative impact on ventilation^112^. At present, we do not know why female eNOS–/– of C57BL6 background in our study had exaggerated responses to HXC, whereas the male eNOS–/– mice did not. We conjecture that the sex difference potentially lies within signaling pathways in carotid body glomus cells and chemoafferent nerve terminals. Finally, it should be mentioned that the male and female WT and eNOS–/– groups all had similar resting body temperatures. This is also in agreement with evidence that resting body temperatures of WT C57BL6 males and females were similar to one another^136^, and that resting body temperatures of eNOS–/– are similar to those of their WT (hybrids of 129/SV and C57BL6 mice) controls^111^.

The presence and distribution of eNOS in the carotid bodies of the mouse (or human) has not been published to date. However, previous publications show that eNOS is richly distributed in several structures in carotid bodies of various species^21,137^. For instance, in the rat, eNOS exists in (1) nerve terminals of carotid sinus chemoafferents, (2) the post-ganglionic sympathetic fibers that emanate from superior cervical ganglia (SCG) and innervate the vasculature within the carotid body, (3) the vascular endothelium itself^21,137–144^, and (4) in some studies, chemosensitive glomus cells of the carotid body^142,143^. Most available evidence suggests that nitric oxide has an inhibitory role in the carotid bodies of all species studied to date. The majority of evidence shows that nitric oxide inhibits glomus cell and chemoafferent nerve responsiveness to hypoxic challenges^145–152^. Nonetheless, there is evidence that nitric oxide has both inhibitory and excitatory effects on cat carotid body chemoreception^153,154^. As such, it is plausible that the lack of eNOS in the carotid bodies of female mice, and the diminished bioavailability of inhibitory nitric oxide during the HXC may partially explain the exaggerated increases in Freq in these mice. The similar responses in other parameters, such as PIF and PEF would suggest that the loss of eNOS outside of the carotid body may not impact the ability of female mice to respond to the HXC.

Another key set of findings from our study is that the post-HXC ventilatory responses that occurred upon return to room-air were markedly reduced in male eNOS–/– mice compared to male WT mice, whereas the responses in female eNOS–/– mice were equivalent to those of female WT mice. It is important to note that the post-HXC responses in the WT female mice were as robust as in the male WT mice. As such, it is clear that the loss of eNOS–/– is not a problem for the female mice, whereas eNOS is absolutely essential for expression of the post-HXC responses in the male mice of C57BL6 background. The post-HXC phase in male eNOS–/– mice was associated with a markedly diminished Freq response (i.e., Freq returned more rapidly and completely to baseline compared to WT control males). The diminished Freq response in the male eNOS–/– mice was the result of a complicated association between Ti and Te. The points to consider include (1) Ti and Te were at baseline levels at 15 min of HXC in male WT and male eNOS–/–mice, (2) Ti dropped dramatically upon return to room-air and took 15 min to return to baseline (in close parallel to the decreases in Freq), (3) Te displayed relatively trivial and short-lasting responses, and (4) the initially diminished Ti in WT and eNOS–/– male mice returned toward baseline equally upon return to room-air (Supplemental Fig. 3), whereas Te rose quite substantially in the eNOS–/– male mice (as opposed to the trivial changes in the WT male mice), and took about 10 min to reach baseline levels (Supplemental Fig. 4). In addition, the post-HXC phase in male eNOS–/– mice was associated with lesser total increases in TV, MV, PIF and PEF, and inspiratory and expiratory drives. It would therefore seem evident that eNOS in male mice is of fundamental importance to the overall return to room-air response after HXC, and thus may play a key role in breathing disorders, including central and obstructive sleep apneas.

The ventilatory responses that occurred during the HXC and upon return to room-air in the female WT mice were as equally pronounced as in their male counterparts. Although the predominant differences in ventilatory responses between WT and eNOS–/– mice were in the males, there were some evident differences between female WT and eNOS–/– mice. These included (a) subtle differences in Freq, Ti, Te and expiratory drive, and more pronounced differences in PIF/PEF in the early stages of the HXC, with a substantially augmented total Te response, and (b) subtle differences in Freq, MV, inspiratory drive and PIF, with more pronounced differences in PEF upon return to room-air. At present, the mechanisms behind these differences in responses in the female eNOS–/– mice remain unknown to us because of the paucity of information of the role of eNOS in the physiology of breathing in female C57BL6 mice. We are currently performing studies using selective inhibitors of eNOS in male and female mice to begin to address the physiological importance of eNOS in relation to breathing and chemoreceptor function.

To our knowledge, there are no existing reports on the role of eNOS during the expression of post-HXC responses in mice or any other species. Considering the generally negative role of nitric oxide in the carotid body, it is difficult to see how the loss of this negative regulator would be responsible for the loss of post-HXC responses in male eNOS–/– mice. Our previous studies showed that the generation of S-nitrosothiols in the carotid bodies and blood may play a pivotal role in the carotid body chemoafferent-mediated increase in ventilation during the post-HXC phase^56,75,155^. This evidence includes that (1) post-HXC ventilatory responses are markedly reduced in mice with bilateral carotid sinus nerve (CSN) transection, and in mice in which red blood hemoglobin cannot generate S-nitrosothiols^56^, (2) post-HXC ventilatory responses are markedly greater in mice lacking S-nitrosogutathione reductase, the enzyme that degrades S-nitrosoglutathione^75^, and (3) arterial injections of L-S-nitrosocysteine cause pronounced increases in minute ventilation via activation of CSN chemoafferents by mechanisms involving activation of voltage-gated K^+^-channels^155^. Whether S-nitrosothiols mediate these CSN-dependent post-HXC responses remains to be established, but it is evident from this study that there is a strong gender-dependent component to the role of eNOS and (potentially S-nitrosothiols) in the ventilatory responses of WT C57BL6 mice.

In summary, our study provides compelling evidence that eNOS plays an important role in the expression of the ventilatory responses to HXC in female, but not male C57BL6 mice, and in the ventilatory responses that occur upon return to room-air in male, but not female C57BL6 mice. The confounding interactions of eNOS and sex in the ventilatory responses of C57BL6 mice before and after HXC are not altogether surprising as it could certainly be expected that the relative role of functional proteins may differ between the sexes. Moreover, the difference in the role of eNOS in C57BL6 mice as opposed to hybrids of 129/SV and C57BL6 mice^111^, support a wealth of evidence as to the importance of neurochemical^86,98–103^ and genetic/sex^86,90–93,96,104–107^ factors in ventilatory signaling and in the responses to hypoxia.

## Supplementary Information

Supplementary Information.
