# Supplementary material for: Short-term facilitation of breathing upon cessation of hypoxic challenge is impaired in male but not female endothelial NOS knock-out mice
Source: Sci Rep. 2021 Sep 15;11:18346. doi: 10.1038/s41598-021-97322-3 (PMC8443732; doi:10.1038/s41598-021-97322-3)
Supplement: Supplementary file 1 — Supplementary Information. [file 41598_2021_97322_MOESM1_ESM.docx]

**Short-term facilitation of breathing upon cessation of hypoxic challenge is impaired in male but not female endothelial NOS knock-out mice**

Paulina M. Getsy,^1,2^ Sripriya Sundararajan,^3,^* Walter J. May,^3^ Graham C. von Schill,^3^

Dylan K. McLaughlin,^3^ Lisa A. Palmer,^3^ Stephen J. Lewis^1,4,5,†^

^1^Department of Pediatrics, Case Western Reserve University, Cleveland, OH, USA

^2^Department of Physiology and Biophysics, Case Western Reserve University, Cleveland, OH, USA

^3^Pediatric Respiratory Medicine, University of Virginia School of Medicine, Charlottesville, VA, USA

^4^Department of Pharmacology, Case Western Reserve University, Cleveland, OH, USA

^5^Functional Electrical Stimulation Center, Case Western Reserve University, Cleveland, OH, USA

***Current Address:** Division of Neonatology, Department of Pediatrics, University of Maryland School of Medicine, Baltimore, Maryland 21201, USA

**^†^Correspondence:** Stephen J. Lewis, PhD. Department of Pediatrics, Biomedical Research Building BRB 319, Case Western Reserve University, 10900 Euclid Avenue Mail Stop 1714, Cleveland, Ohio 44106-1714. Email: sjl78@case.edu

**Acknowledgments**

The authors wish to thank the staff at the Animal Care Facilities at the University of Virginia. The authors also wish to acknowledge Ms. Chelsea Csuhran and Ms. Yvonnda West for their important contribution to data collection and data analyses.

**Abbreviated Title (Running Head):** Hypoxic response in eNOS knockout mice

**Supplemental Figure 1**

**A.**

**B.**

**C.**

**Supplementary Figure 1.** **Panel A:** Frequency of breathing (Freq), **Panel B:** Tidal volume, and **Panel C:** Minute ventilation values before, during a hypoxic gas challenge (HXC, 10% O_2_, 90% N_2_), and upon return to room-air in male (M) wild-type (WT) and eNOS knock-out (eNOS-/-) male mice. The data are presented as mean ± SD.

**Supplemental Figure 2**

**A.**

**B.**

**C.**

**D.**

**E.**

**F.**

**Supplemental Figure 2.** **Panels A, C and E:** Arithmetic changes in frequency (Fr), tidal volume (TV) and minute ventilation (MV) in WT and eNOS-/- male mice during the first 90 sec of exposure to the HXC and the first 90 sec upon return to room-air. **Panels B, D and F:** Total changes in frequency (Freq), TV and MV in WT and eNOS-/- male mice during the HXC and during the first 5 min (RA5) and entire 15 min (RA15) return to room-air. The data are shown as mean ± SD. The data were analyzed by one-way or two-way ANOVA followed by Student’s modified t-test with Bonferroni corrections for multiple comparisons between means using the error mean square terms from each ANOVA. **P* < 0.05, significant response. **^†^***P* < 0.05, eNOS-/- *versus* WT within the male sex.

**Supplemental Figure 3**

**B.**

**A.**

**C.**

**D.**

**E.**

**Supplemental Figure 3.** **Panels A and B:** Inspiratory time (Ti) values before, during a hypoxic gas challenge (HXC, 10% O_2_, 90% N_2_), and upon return to room-air in male (M) and female (F) wild-type (WT) and eNOS knock-out (eNOS-/-) mice. P**anels C and D:** Arithmetic changes in Ti in WT and eNOS-/- male and female mice during the first 90 sec of exposure to the HXC and the first 90 sec upon return to room-air. **Panel E:** Total changes in Ti in WT and eNOS-/- male and female mice during HXC and during the first 5 min (RA5) and entire 15 min (RA15) return to room-air. The data are shown as mean ± SEM. The data were analyzed by one-way or two-way ANOVA followed by Student’s modified t-test with Bonferroni corrections for multiple comparisons between means using the error mean square terms from each ANOVA. *P < 0.05, significant response. **^†^**P < 0.05, eNOS-/- *versus* WT within each sex.

**Supplemental Figure 4**

**B.**

**A.**

**D.**

**C.**

**E.**

**Supplemental Figure 4.** **Panels A and B:** Expiratory time (Te) values before, during a hypoxic gas challenge (HXC, 10% O_2_, 90% N_2_), and upon return to room-air in male (M) and female (F) wild-type (WT) and eNOS knock-out (eNOS-/-) mice. **Panels C and D:** Arithmetic changes in Te in WT and eNOS-/- male and female mice during the first 90 sec of exposure to the HXC and the first 90 sec upon return to room-air. **Panel E:** Total changes in Te in WT and eNOS-/- male and female mice during HXC and during the first 5 min (RA5) and entire 15 min (RA15) return to room-air. The data are shown as mean ± SEM. The data were analyzed by one-way or two-way ANOVA followed by Student’s modified t-test with Bonferroni corrections for multiple comparisons between means using the error mean square terms from each ANOVA. *P < 0.05, significant response. **^†^**P < 0.05, eNOS-/- *versus* WT within each sex.

**Supplemental Figure 5**

**B.**

**A.**

**D.**

**C.**

**E.**

**Supplemental Figure 5.** **Panels A and B:** Peak Inspiratory Flow (PIF) values before, during a hypoxic gas challenge (HXC, 10% O_2_, 90% N_2_), and upon return to room-air in male (M) and female (F) wild-type (WT) and eNOS knock-out (eNOS-/-) mice. **Panels C and D:** Arithmetic changes in PIF in WT and eNOS-/- male and female mice during the first 90 sec of exposure to the HXC and the first 90 sec upon return to room-air. **Panel** **E:** Total changes in PIF in WT and eNOS-/- male and female mice during HXC and during the first 5 min (RA5) and entire 15 min (RA15) return to room-air. The data are shown as mean ± SEM. The data were analyzed by one-way or two-way ANOVA followed by Student’s modified t-test with Bonferroni corrections for multiple comparisons between means using the error mean square terms from each ANOVA. *P < 0.05, significant response. **^†^**P < 0.05, eNOS-/- *versus* WT within each sex.

**Supplemental Figure 6**

**A.**

**B.**

**D.**

**C.**

**E.**

**Supplemental Figure 6.** **Panels A and B:** Peak Expiratory Flow (PEF) (Peak Exp Flow) values before, during a hypoxic gas challenge (HXC, 10% O_2_, 90% N_2_) and upon return to room-air in male (M) and female (F) wild-type (WT) and eNOS knock-out (eNOS-/-) mice. **Panels C and D:** Arithmetic changes in PEF in WT and eNOS-/- male and female mice during the first 90 sec of exposure to the HXC and the first 90 sec upon return to room-air. **Panel** **E:** Total changes in PEF in WT and eNOS-/- male and female mice during HXC and during the first 5 min (RA5) and entire 15 min (RA15) return to room-air. Data are shown as mean ± SEM. The data were analyzed by one-way or two-way ANOVA followed by Student’s modified t-test with Bonferroni corrections for multiple comparisons between means using the error mean square terms from each ANOVA. *P < 0.05, significant response. **^†^**P < 0.05, eNOS-/- *versus* WT within each sex.
